# Supplementary material for: Changes in non-communicable diseases, diet and exercise in a rural Bangladesh setting before and after the first wave of COVID-19
Source: PLOS Glob Public Health. 2022 Sep 30;2(9):e0001110. doi: 10.1371/journal.pgph.0001110 (PMC10021158; doi:10.1371/journal.pgph.0001110)
Supplement: S1 File — Appendix A. Summary of survey weighting. Appendix B. Anthropometric and mental health measures, by gender. Appendix C. Anthropometric and mental health measures, by age group. Appendix D. Anthropometric and mental health measures, by wealth group. (DOCX) [file pgph.0001110.s001.docx]

**Appendix A: Survey weighting**

| **Cluster** | **Survey 1**  **N (mean weight)** | **Survey 2**  **N (mean weight)** |
| --- | --- | --- |
| Alfadanga 1 | 75 (1.00) | 121 (0.99) |
| Alfadanga 2 | 94 (1.13) | 111 (1.07) |
| Bana 1 | 74 (1.00) | 114 (1.08) |
| Bana 2 | 96 (1.01) | 118 (0.95) |
| Buraich 1 | 97 (1.20) | 115 (1.11) |
| Buraich 2 | 64 (0.92) | 115 (0.90) |
| Golpalpur 1 | 92 (0.97) | 107 (0.95) |
| Golpalpur 2 | 63 (0.97) | 117 (0.91) |
| Panchuria 1 | 84 (0.86) | 122 (0.84) |
| Panchuria 2 | 69 (1.37) | 125 (1.28) |
| Tagarbanda 1 | 88 (0.91) | 113 (0.99) |
| Tagarbanda 2 | 54 (0.71) | 114 (0.73) |

**Appendix B: Anthropometric and mental health measures, by gender**

|  | | **Men** | | | | | | | **Women** | | | | | | |
| --- | --- | --- | --- | --- | --- | --- | --- | --- | --- | --- | --- | --- | --- | --- | --- |
| **Condition** | | **Survey 1 (n=372)** | | | **Survey 2 (n=516)** | | | **p-value** | **Survey 1 (n=576)** | | | **Survey 2 (n=876)** | | | **p-value** |
|  |  | **N** | **% (95% CI)** | | **N** | **% (95% CI)** | |  | **N** | **% (95% CI)** | | **N** | **% (95% CI)** | |  |
| Diabetes | Normal | 234 | 62.3% | (55.4, 68.8) | 316 | 59.2% | (53.6, 64.7) | 0.493 | 315 | 54.7% | (49.1, 60.1) | 495 | 57.1% | (51.0, 63.0) | 0.540 |
|  | IGF | 18 | 4.8% | (2.2, 10.6) | 31 | 6.8% | (4.7, 9.8) |  | 23 | 3.1% | (1.8, 5.1) | 33 | 4.1% | (3.0, 5.6) |  |
|  | IGT | 69 | 18.9% | (15.3, 23.2) | 83 | 16.9% | (13.7, 20.6) |  | 134 | 23.5% | (20.2, 27.2) | 179 | 20.5% | (16.0, 25.8) |  |
|  | T2DM | 48 | 13.9% | (10.9, 17.5) | 86 | 17.1% | (12.7, 22.7) |  | 104 | 18.7% | (15.7, 22.3) | 164 | 18.4% | (15.3, 21.8) |  |
| Hypertension | | 100 | 30.9% | (25.7, 36.5) | 188 | 40.8% | (35.8, 45.9) | 0.012 | 192 | 37.2% | (32.3, 42.3) | 349 | 42.0% | (37.7, 46.5) | 0.148 |
| BMI | Underweight | 68 | 18.3% | (14.2, 23.3) | 71 | 15.0% | (11.2, 19.8) | 0.583 | 46 | 8.2% | (6.4, 10.3) | 91 | 11.5% | (9.3, 14.1) | 0.291 |
|  | Normal | 179 | 47.8% | (43.4, 52.2) | 253 | 48.0% | (43.7, 52.4) |  | 212 | 37.9% | (33.8, 42.2) | 309 | 36.1% | (32.6, 39.8) |  |
|  | Overweight | 103 | 28.0% | (23.8, 32.6) | 163 | 31.5% | (25.6, 37.9) |  | 241 | 41.4% | (36.0, 47.1) | 358 | 39.3% | (35.4, 43.3) |  |
|  | Obese | 19 | 5.9% | (3.5, 9.7) | 28 | 5.5% | (3.4, 8.7) |  | 75 | 12.5% | (9.6, 16.1) | 118 | 13.2% | (10.4, 16.5) |  |
| Abdominal obesity | | 223 | 59.5% | (52.7, 66.0) | 337 | 65.1% | (56.1, 73.1) | 0.308 | 377 | 65.5% | (58.6, 71.9) | 602 | 67.7% | (59.9, 74.7) | 0.655 |
| Depression | | 32 | 8.5% | (3.5, 19.1) | 23 | 4.8% | (2.0, 10.7) | 0.326 | 121 | 20.1% | (12.0, 31.8) | 61 | 6.8% | (3.1, 14.3) | 0.016 |
| Anxiety | | 45 | 12.1% | (6.5, 21.6) | 13 | 2.5% | (1.0, 6.3) | 0.004 | 132 | 22.1% | (14.9, 31.4) | 47 | 4.9% | (2.7, 8.9) | <0.001 |
|  | | **Mean** | **(95% CI)** | | **Mean** | **(95% CI)** | | **p-value** | **Mean** | **(95% CI)** | | **Mean** | **(95% CI)** | | **p-value** |
| Fasting blood glucose | | 5.72 | (5.41, 6.02) | | 5.82 | (5.50, 6.13) | | 0.638 | 5.95 | (5.70, 6.20) | | 5.89 | (5.69, 6.10) | | 0.711 |
| Systolic blood pressure | | 127.55 | (124.78, 130.31) | | 135.75 | (133.80, 137.69) | | <0.001 | 127.31 | (124.99, 129.63) | | 133.27 | (130.75, 135.78) | | 0.002 |
| Diastolic blood pressure | | 73.30 | (71.22, 75.37) | | 78.51 | (77.40, 79.61) | | <0.001 | 72.74 | (71.37, 74.12) | | 76.71 | (74.92, 78.49) | | 0.001 |
| BMI | | 21.80 | (21.29, 22.30) | | 22.04 | (21.43, 22.66) | | 0.528 | 23.60 | (23.18, 24.02) | | 23.43 | (23.09, 23.78) | | 0.535 |
| Waist to hip ratio | | 0.92 | (0.91, 0.93) | | 0.92 | (0.91, 0.93) | | 0.691 | 0.88 | (0.87, 0.89) | | 0.88 | (0.86, 0.89) | | 0.800 |
| Self-rated health | | 74.72 | (70.65, 78.79) | | 84.21 | (79.73, 88.68) | | 0.004 | 68.90 | (63.55, 74.26) | | 79.17 | (74.00, 84.34) | | 0.009 |
| PHQ-9 score | | 2.36 | (1.35, 3.37) | | 1.46 | (0.80, 2.11) | | 0.135 | 3.93 | (2.69, 5.18) | | 2.10 | (1.18, 3.03) | | 0.023 |
| GAD-7 score | | 4.95 | (3.82, 6.08) | | 2.79 | (2.28, 3.29) | | 0.001 | 6.29 | (5.26, 7.32) | | 4.08 | (3.25, 4.90) | | 0.002 |

**Appendix C: Anthropometric and mental health measures, by age group**

|  | | **<60 years old** | | | | | | | **>= 60 years old** | | | | | | |
| --- | --- | --- | --- | --- | --- | --- | --- | --- | --- | --- | --- | --- | --- | --- | --- |
| **Condition** | | **Survey 1 (n=709)** | | | **Survey 2 (n=1010)** | | | **p-value** | **Survey 1 (n=239)** | | | **Survey 2 (n=382)** | | | **p-value** |
|  |  | **N** | **% (95% CI)** | | **N** | **% (95% CI)** | |  | **N** | **% (95% CI)** | | **N** | **% (95% CI)** | |  |
| Diabetes | Normal | 425 | 59.2% | (53.1, 65.0) | 600 | 59.0% | (53.3, 64.5) | 0.328 | 124 | 54.3% | (47.4, 61.1) | 211 | 55.5% | (48.7, 62.0) | 0.782 |
|  | IGF | 29 | 3.6% | (2.0, 6.7) | 29 | 5.0% | (3.5, 7.2) |  | 12 | 4.3% | (2.7, 6.8) | 17 | 5.5% | (3.0, 9.9) |  |
|  | IGT | 149 | 22.1% | (19.0, 25.6) | 183 | 18.4% | (14.8, 22.6) |  | 54 | 20.2% | (16.3, 24.7) | 79 | 20.6% | (16.3, 25.7) |  |
|  | T2DM | 104 | 15.0% | (12.8, 17.5) | 178 | 17.6% | (14.1, 21.7) |  | 48 | 21.2% | (15.8, 27.9) | 72 | 18.5% | (14.7, 22.9) |  |
| Hypertension | | 166 | 25.9% | (21.7, 30.5) | 298 | 31.3% | (28.2, 34.7) | 0.055 | 126 | 57.4% | (50.9, 63.7) | 239 | 65.5% | (59.6, 70.9) | 0.064 |
| BMI | Underweight | 58 | 8.1% | (5.7, 11.4) | 87 | 8.9% | (7.0, 11.4) | 0.762 | 56 | 24.1% | (20.2, 28.5) | 75 | 21.9% | (18.4, 25.9) | 0.354 |
|  | Normal | 275 | 40.1% | (36.3, 44.1) | 391 | 38.9% | (36.6, 41.3) |  | 116 | 47.2% | (42.0, 52.6) | 171 | 45.2% | (40.4, 50.0) |  |
|  | Overweight | 292 | 40.7% | (36.9, 44.6) | 404 | 39.6% | (36.0, 43.2) |  | 52 | 22.6% | (16.1, 30.7) | 117 | 28.4% | (23.9, 33.5) |  |
|  | Obese | 82 | 11.1% | (8.5, 14.4) | 127 | 12.6% | (10.0, 15.7) |  | 12 | 6.1% | (3.5, 10.3) | 19 | 4.4% | (2.8, 7.0) |  |
| Abdominal obesity | | 456 | 63.8% | (58.3, 69.0) | 681 | 67.0% | (60.5, 73.0) | 0.432 | 144 | 60.8% | (53.9, 67.4) | 258 | 65.8% | (58.2, 72.7) | 0.311 |
| Depression | | 100 | 12.9% | (6.7, 23.2) | 38 | 3.5% | (1.4, 8.3) | 0.014 | 53 | 21.5% | (12.2, 35.3) | 46 | 11.6% | (5.5, 22.9) | 0.161 |
| Anxiety | | 134 | 17.6% | (10.7, 27.6) | 36 | 3.4% | (1.6, 7.0) | <0.001 | 43 | 18.6% | (11.4, 29.0) | 24 | 5.3% | (2.4, 11.3) | 0.006 |
|  | | **Mean** | **(95% CI)** | | **Mean** | **(95% CI)** | | **p-value** | **Mean** | **(95% CI)** | | **Mean** | **(95% CI)** | | **p-value** |
| Fasting blood glucose | | 5.78 | (5.51, 6.05) | | 5.89 | (5.64, 6.14) | | 0.555 | 6.05 | (5.73, 6.36) | | 5.81 | (5.59, 6.02) | | 0.210 |
| Systolic blood pressure | | 123.33 | (120.86, 125.79) | | 128.78 | (127.04, 130.52) | | 0.001 | 138.28 | (135.07, 141.49) | | 146.82 | (143.87, 149.77) | | <0.001 |
| Diastolic blood pressure | | 72.74 | (71.31, 74.16) | | 76.97 | (75.67, 78.27) | | <0.001 | 73.61 | (71.42, 75.79) | | 78.46 | (76.35, 80.56) | | 0.003 |
| BMI | | 23.37 | (23.14, 23.61) | | 23.48 | (23.12, 23.84) | | 0.613 | 21.42 | (20.87, 21.96) | | 21.51 | (21.10, 21.92) | | 0.782 |
| Waist to hip ratio | | 0.89 | (0.89, 0.90) | | 0.89 | (0.88, 0.90) | | 0.709 | 0.90 | (0.89, 0.91) | | 0.90 | (0.89, 0.91) | | 0.799 |
| Self-rated health | | 74.07 | (69.37, 78.78) | | 83.63 | (79.01, 88.25) | | 0.006 | 64.09 | (59.04, 69.14) | | 75.51 | (69.89, 81.13) | | 0.005 |
| PHQ-9 score | | 2.89 | (1.89, 3.89) | | 1.46 | (0.88, 2.03) | | 0.017 | 4.29 | (2.67, 5.91) | | 2.74 | (1.28, 4.20) | | 0.154 |
| GAD-7 score | | 5.56 | (4.48, 6.65) | | 3.34 | (2.62, 4.06) | | 0.002 | 6.17 | (5.35, 6.99) | | 4.08 | (3.38, 4.78) | | 0.001 |

**Appendix D: Anthropometric and mental health measures, by wealth group**

|  | | | **Poorest** | | | | | | | **Middle** | | | | | | | **Wealthiest** | | | | | | |
| --- | --- | --- | --- | --- | --- | --- | --- | --- | --- | --- | --- | --- | --- | --- | --- | --- | --- | --- | --- | --- | --- | --- | --- |
| **Condition** | | | **Survey 1 (n=332)** | | | **Survey 2 (n=445)** | | | **p-value** | **Survey 1 (n=316)** | | | **Survey 2 (n=460)** | | | **p-value** | **Survey 1 (n=297)** | | | **Survey 2 (n=482)** | | | **p-value** |
|  |  |  | **N** | **% (95% CI)** | | **N** | **% (95% CI)** | |  | **N** | **% (95% CI)** | | **N** | **% (95% CI)** | |  | **N** | **% (95% CI)** | | **N** | **% (95% CI)** | |  |
| Diabetes | | Normal | 203 | 61.2% | (54.7, 67.3) | 273 | 60.5% | (53.8, 66.9) | 0.996 | 190 | 61.7% | (56.5, 66.6) | 282 | 61.2% | (55.2, 67.0) | 0.119 | 156 | 50.5% | (44.6, 56.5) | 256 | 52.4% | (46.4, 58.4) | 0.821 |
|  | | IGF | 15 | 4.2% | (2.4, 7.4) | 19 | 4.5% | (2.9, 7.0) |  | 13 | 3.0% | (1.5, 5.9) | 23 | 5.7% | (3.6, 8.8) |  | 13 | 4.3% | (1.9, 9.5) | 22 | 5.3% | (3.6, 7.6) |  |
|  | | IGT | 67 | 20.6% | (15.1, 27.5) | 88 | 20.6% | (15.7, 26.5) |  | 70 | 21.7% | (18.0, 25.9) | 81 | 16.9% | (14.1, 20.0) |  | 66 | 22.6% | (18.2, 27.7) | 93 | 19.8% | (15.0, 25.5) |  |
|  | | T2DM | 47 | 14.0% | (10.0, 19.4) | 65 | 14.3% | (10.8, 18.8) |  | 43 | 13.7% | (10.5, 17.7) | 74 | 16.3% | (12.5, 20.8) |  | 62 | 22.6% | (17.3, 29.0) | 111 | 22.5% | (19.0, 26.5) |  |
| Hypertension | | | 93 | 31.7% | (26.2, 37.8) | 166 | 38.9% | (35.2, 42.8) | 0.044 | 89 | 31.0% | (26.2, 36.2) | 157 | 37.1% | (34.8, 39.4) | 0.037 | 109 | 40.5% | (32.4, 49.1) | 214 | 48.1% | (39.3, 57.0) | 0.219 |
| BMI | Underweight | | 50 | 16.1% | (11.8, 21.7) | 77 | 17.8% | (15.0, 21.0) | 0.577 | 42 | 14.6% | (11.1, 19.2) | 48 | 12.0% | (7.7, 18.1) | 0.800 | 22 | 6.4% | (4.2, 9.5) | 37 | 9.3% | (7.3, 11.9) | 0.322 |
|  | Normal | | 155 | 47.0% | (43.2, 50.8) | 207 | 47.7% | (43.2, 52.3) |  | 132 | 42.2% | (36.7, 47.8) | 200 | 42.9% | (39.7, 46.1) |  | 104 | 37.0% | (31.8, 42.5) | 155 | 32.8% | (27.3, 38.7) |  |
|  | Overweight | | 106 | 31.3% | (26.6, 36.5) | 131 | 27.6% | (24.1, 31.4) |  | 112 | 34.3% | (28.0, 41.3) | 170 | 35.6% | (29.0, 42.8) |  | 126 | 41.9% | (38.3, 45.6) | 220 | 44.4% | (38.7, 50.2) |  |
|  | Obese | | 20 | 5.6% | (3.5, 8.9) | 30 | 6.9% | (4.8, 9.7) |  | 30 | 8.9% | (6.4, 12.2) | 45 | 9.6% | (6.3, 14.4) |  | 44 | 14.8% | (10.6, 20.3) | 71 | 13.5% | (10.6, 17.0) |  |
| Abdominal obesity | | | 186 | 54.4% | (49.2, 59.6) | 268 | 60.6% | (52.1, 68.5) | 0.213 | 200 | 61.8% | (52.4, 70.4) | 309 | 65.0% | (57.4, 71.9) | 0.578 | 214 | 73.0% | (65.1, 79.6) | 362 | 73.8% | (68.3, 78.6) | 0.854 |
| Depression | | | 59 | 16.6% | (9.4, 27.6) | 34 | 6.6% | (2.8, 14.9) | 0.060 | 47 | 13.7% | (7.0, 25.1) | 26 | 6.3% | (2.9, 13.4) | 0.120 | 47 | 15.6% | (7.7, 29.2) | 24 | 5.1% | (2.1, 11.7) | 0.036 |
| Anxiety | | | 66 | 18.6% | (11.7, 28.2) | 20 | 3.9% | (1.8, 8.1) | <0.001 | 60 | 18.5% | (10.8, 29.9) | 18 | 3.8% | (1.6, 8.7) | 0.001 | 51 | 16.7% | (9.1, 28.6) | 22 | 4.2% | (2.2, 7.8) | 0.002 |
|  | | | **Mean** | **(95% CI)** | | **Mean** | **(95% CI)** | |  | **Mean** | **(95% CI)** | | **Mean** | **(95% CI)** | | **p-value** | **Mean** | **(95% CI)** | | **Mean** | **(95% CI)** | | **p-value** |
| Fasting blood glucose | | | 5.58 | (5.34, | 5.82) | 5.49 | (5.20, | 5.79) | 0.633 | 5.75 | (5.42, 6.08) | | 5.80 | (5.57, 6.02) | | 0.803 | 6.24 | (5.89, 6.58) | | 6.26 | (6.00, 6.54) | | 0.933 |
| Systolic blood pressure | | | 127.1 | (124.3 | 129.9) | 133.4 | (131.4, | 135.4) | 0.001 | 125.8 | (122.7, 128.9) | | 132.4 | (130.0, 134.9) | | 0.002 | 129.4 | (126.5, 132.3) | | 136.8 | (133.0, 140.5) | | 0.004 |
| Diastolic blood pressure | | | 72.5 | (70.6, | 74.3) | 76.9 | (75.8, | 78.0) | <0.001 | 72.1 | (70.0, 74.3) | | 76.1 | (74.4, 77.8) | | 0.007 | 74.4 | (72.7, 76.0) | | 79.2 | (77.3, 81.0) | | 0.001 |
| BMI | | | 22.0 | (21.5, | 22.5) | 21.9 | (21.6, | 22.2) | 0.736 | 22.6 | (22.1, 23.0) | | 22.8 | (22.2, 23.4) | | 0.503 | 24.0 | (23.5, 24.5) | | 23.8 | (23.4, 24.3) | | 0.615 |
| Waist to hip ratio | | | 0.88 | (0.87, | 0.89) | 0.88 | (0.87, | 0.89) | 0.969 | 0.89 | (0.88, 0.90) | | 0.89 | (0.88, 0.90) | | 0.958 | 0.91 | (0.90, 0.92) | | 0.91 | (0.89, 0.92) | | 0.604 |
| Self-rated health | | | 67.3 | (62.2, | 72.4) | 81.9 | (75.9, | 87.8) | 0.001 | 73.3 | (69.1, 77.5) | | 81.0 | (76.4, 85.5) | | 0.018 | 73.3 | (68.4, 78.2) | | 80.8 | (76.0, 85.6) | | 0.034 |
| PHQ-9 score | | | 3.30 | (2.30, | 4.30) | 2.08 | (1.13, | 3.02) | 0.079 | 3.18 | (1.87, 4.49) | | 1.90 | (0.94, 2.87) | | 0.120 | 3.35 | (2.00, 4.71) | | 1.59 | (0.87, 2.31) | | 0.026 |
| GAD-7 score | | | 5.74 | (4.65, | 6.83) | 3.92 | (3.05, | 4.80) | 0.013 | 5.86 | (4.61, 7.11) | | 3.57 | (2.77, 4.37) | | 0.004 | 5.58 | (4.65, 6.51) | | 3.25 | (2.68, 3.81) | | <0.001 |
