# Supplementary material for: Changes in non-communicable diseases, diet and exercise in a rural Bangladesh setting before and after the first wave of COVID-19
Source: PLOS Glob Public Health. 2022 Sep 30;2(9):e0001110. doi: 10.1371/journal.pgph.0001110 (PMC10021158; doi:10.1371/journal.pgph.0001110)
Supplement: Attachment — Submitted filename: ReviewerResponse_WP2CovidPaper.docx [file pgph.0001110.s003.docx]

**Editorial Comments:**

1. In the hypertension definition, did the authors consider those as hypertensives, who were taking antihypertensive medications but had normal blood pressure?

*Thank you for noticing this - yes, our binary variable for hypertension included both those who met the definition based on their blood pressure measurement, taken by study staff on the day of the survey, AND respondents who self-reported that they had been diagnosed with hypertension by a medical professional. We have clarified this in Table 1.*

2. Please add a supplementary table to describe the weighting methods.

*This has been added as Supplementary Appendix 1 (and other appendices re-labelled).*

3. In the tables, are the frequencies and percentages weighted or unweighted? Please mention that in the footnote.

*All the information in the analysis tables (3 - 5) is based on the weighted data. We have added this into the footnotes as requested.*

**Reviewer #1:**

Thank you for submitting this well-written manuscript. I believe it will be a good addition to the global pool of literature. Hence, I am happy to recommend it for publication.

*Thank you for the positive review of our paper!*

**Reviewer #2:**

The concept of this manuscript is good. Two issues should be taken into consideration.

*Thank you for the positive review of our paper!*

1. First of all graphical description of some analysis is more suitable. For example, if the increasing rate of hypertension or the lower prevalence of depression and anxiety could be presented via some plots that would be better.

*Thank you for this suggestion – we had plotted box and whisker diagrams and decided to remove them before submission. We’ve now happily added these back into the paper as Figures 2 and 3!*

2. The second one is about the improvement in mental health on the second survey. This finding contradicts the typical scenario of the whole world at that time. The reason provided behind it does not seem to be so strong. It needs to be reviewed again.

*We agree, this finding was surprising to us, and we spent some time discussing as a team to hypothesise why self-reported measures of health and wellbeing improved. While we had plausible theories, finding other published literature that used representative population samples, and measured wellbeing and mental health after (instead of during) periods of COVID-19 restrictions was difficult. Therefore, we tried to be careful to not over-state our hypotheses. We have gone back through this section of the discussion, to make it clearer that these findings are novel, but we are unable to fully explain them (pg. 15-16). As with much of the narratives around mental health and COVID-19 prevention interventions, there is clearly a lot more contextual nuance that needs to be unpacked!*

**Journal Requirements**

1. Please include a complete copy of PLOS’ questionnaire on inclusivity in global research in your revised manuscript. Our policy for research in this area aims to improve transparency in the reporting of research performed outside of researchers’ own country or community. The policy applies to researchers who have travelled to a different country to conduct research, research with Indigenous populations or their lands, and research on cultural artefacts. The questionnaire can also be requested at the journal’s discretion for any other submissions, even if these conditions are not met.  Please find more information on the policy and a link to download a blank copy of the questionnaire here: <https://journals.plos.org/globalpublichealth/s/best-practices-in-research-reporting>. Please upload a completed version of your questionnaire as Supporting Information when you resubmit your manuscript.

*Now completed and uploaded as a supplementary file “Ethics Checklist” and referenced in the methods as suggested.*

2. We noticed that you used "unpublished data" in the manuscript. We do not allow these references, as the PLOS data access policy requires that all data be either published with the manuscript or made available in a publicly accessible database. Please either remove these references, or amend the supplementary material to include the referenced data.

*We have removed this sentence – the manuscript with this information is currently under-review, and so not ready to reference.*

3. Please update the completed 'Competing Interests' statement, including any COIs declared by your co-authors. If you have no competing interests to declare, please state "The authors have declared that no competing interests exist".

*We confirm that the authors have no competing interests, and have updated this statement (pg 19).*

4. We have noticed that you have uploaded supporting information but you have not included a list of legends.  Please add a full list of legends for all supporting information files (including figures, table and data files) after the references list.

*A legend for the supporting information is now included on page 23.*

5. In the online submission form, you indicated that "These data are part of an on-going randomised controlled trial and will be made openly accessible once the main trial results have been published (expected early 2023). In the meantime, data will be made available upon reasonable request for research purposed only. Please contact Prof Ed Fottrell as the Principal Investigator ([e.fottrell@ucl.ac.uk](mailto:e.fottrell@ucl.ac.uk)).". All PLOS journals now require all data underlying the findings described in their manuscript to be freely available to other researchers, either 1. In a public repository, 2. Within the manuscript itself, or 3. Uploaded as supplementary information. This policy applies to all data except where public deposition would breach compliance with the protocol approved by your research ethics board. If your data cannot be made publicly available for ethical or legal reasons (e.g., public availability would compromise patient privacy), please explain your reasons by return email and your exemption request will be escalated to the editor for approval. Your exemption request will be handled independently and will not hold up the peer review process, but will need to be resolved should your manuscript be accepted for publication. One of the Editorial team will then be in touch if there are any issues.

*We have now uploaded a fully anonymised dataset in Figshare as a public repository (*[*https://doi.org/10.6084/m9.figshare.19337831.v1*](https://doi.org/10.6084/m9.figshare.19337831.v1)*), after reflecting on whether this would have negative consequences for the conduct of the main trial.*

6. Please amend your detailed Financial Disclosure statement. This is published with the article, therefore should be completed in full sentences and contain the exact wording you wish to be published. ii). State the initials, alongside each funding source, of each author to receive each grant. iii). State what role the funders took in the study. If the funders had no role in your study, please state: “The funders had no role in study design, data collection and analysis, decision to publish, or preparation of the manuscript.”

*This has been updated.*
