# Supplementary material for: Changes in non-communicable diseases, diet and exercise in a rural Bangladesh setting before and after the first wave of COVID-19
Source: PLOS Glob Public Health. 2022 Sep 30;2(9):e0001110. doi: 10.1371/journal.pgph.0001110 (PMC10021158; doi:10.1371/journal.pgph.0001110)
Supplement: Attachment — Submitted filename: ReviewerResponse_WP2CovidPaper_R2.docx [file pgph.0001110.s004.docx]

Reviewer #4: Thank you for conducting this study and submitting it to PLOS Global Health! Overall, this study certainly is very rigorous and contains rich data, insights, and analysis. However, I have a few minor comments below addressing which, I believe, would further enrich the study.

1. Apparently you have conducted your study in a Upazilla/sub-district of Faridpur district. Given there are 492 Upazillas in total, I wonder if the results from one Upazilla could be generalized to the entire rural Bangladesh. Furthermore, I wonder if this specific sub-district was chosen due to convenience of data collection or due to any other decision criteria. It would be great if you could present your case as to why you believe the results are generalizable – or, maybe change the title of the study accordingly.

***Alfadanga upazila was purposefully chosen for the larger D:Clare for mainly practical reasons, which are specified in the trial protocol paper, but we have now elaborated in the text. While the choice was more pragmatic, the socioeconomic make-up of the population is typical of rural Bangladesh and our random sampling approach should mean our findings are more generalizable. However, we appreciate that localised policies, COVID-19 epidemiology and behavioural factors may vary across rural Bangladesh. We have edited the title and re-read to ensure we are not making generalisations that are too broad.***

2. Line 97-99: It would be easier for the readers and reviewers to understand if you could explain the rationale behind setting up those criteria e.g. eligible villages were those which do not sit on a border with a neighbouring study cluster etc.

***The villages were selected purposefully within study clusters using a “fried egg” approach to try and minimise contamination between intervention and control clusters for the main trial. We do not expect that villages in the centre of clusters would differ from villages on the borders of our cluster areas, as the clusters were based on administrative units (unions) which were then split into two clusters based on approximate population size. We have added some additional information to the manuscript (line 99).***

3. Line 117-120: Was any form of monetary/non-monetary remuneration provided to the participants?

***We did not provide any form of incentive or remuneration for participation and have added this to the text (line 124).***

4. Line 141-152: Did you have any missing data? If so, would you please describe how you had dealt with missing data in your analysis?

***We had stated in the analysis section that we used a complete case analysis approach as the missing data was very limited – we had just two participants in survey 1 who did not complete the anthropometry data collection and two who did not complete the survey, otherwise there was no missing data, and these are indicated in the table footnotes.***

5. The inclusion of self-reported diagnosis of hypertension by a healthcare provider as one of the measures of hypertension yields the possibility of non-differential misclassification bias and recall bias. It could be useful to acknowledge that in the discussion/limitation section.

***This is a good point – we have added this to the limitations (line 332).***
